# Monitoring of Apoptosis in 3D Cell Cultures by FRET and Light Sheet Fluorescence Microscopy

**DOI:** 10.3390/ijms16035375

**Published:** 2015-03-09

**Authors:** Petra Weber, Sarah Schickinger, Michael Wagner, Brigitte Angres, Thomas Bruns, Herbert Schneckenburger

**Affiliations:** 1 Institute of Applied Research, Aalen University, Anton-Huber Str. 21, 73430 Aalen, Germany; E-Mails: petra.weber@htw-aalen.de (P.W.); sarah.schickinger@htw-aalen.de (S.S.); michael.wagner@htw-aalen.de (M.W.); thomas.bruns@htw-aalen.de (T.B.); 2 Cellendes GmbH, Markwiesenstr. 55, 72770 Reutlingen, Germany; E-Mail: brigitte.angres@cellendes.com

**Keywords:** energy transfer, FRET, apoptosis, light sheet fluorescence microscopy (LSFM), single plane fluorescence microscopy (SPIM), fluorescence lifetime imaging (FLIM), microspectral analysis, cell spheroids

## Abstract

Non-radiative cell membrane associated Förster Resonance Energy Transfer (FRET) from an enhanced cyan fluorescent protein (ECFP) to an enhanced yellow fluorescent protein (EYFP) is used for detection of apoptosis in 3-dimensional cell cultures. FRET is visualized in multi-cellular tumor spheroids by light sheet based fluorescence microscopy in combination with microspectral analysis and fluorescence lifetime imaging (FLIM). Upon application of staurosporine and to some extent after treatment with phorbol-12-myristate-13-acetate (PMA), a specific activator of protein kinase c, the caspase-3 sensitive peptide linker DEVD is cleaved. This results in a reduction of acceptor (EYFP) fluorescence as well as a prolongation of the fluorescence lifetime of the donor (ECFP). Fluorescence spectra and lifetimes may, therefore, be used for monitoring of apoptosis in a realistic 3-dimensional system, while light sheet based microscopy appears appropriate for 3D imaging at low light exposure.

## 1. Introduction

**Figure 1:**
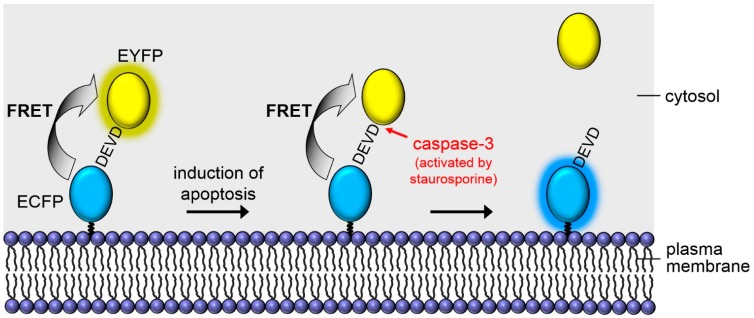
Cell membrane associated Förster Resonance Energy Transfer (FRET)-based sensor system for apoptosis.

In a previous paper on single cells and cell monolayers [1] we showed that cell membrane associated Förster Resonance Energy Transfer (FRET) [2] from a cyan to a yellow fluorescent protein can be used for measurement of apoptosis. Enhanced cyan fluorescent protein (ECFP) was anchored in the plasma membrane of the cells, and via the caspase-3 sensitive peptide linker “Aspartic acid–Glutamic acid–Valine–Aspartic acid” (1-Letter-code: DEVD) [3,4] bound to enhanced yellow fluorescent protein (EYFP). Non-resonant energy transfer from ECFP to EYFP was observed, but interrupted in the case of apoptosis when the DEVD linker was cleaved, as visualized in Figure 1. Therefore, the Mem-ECFP-DEVD-EYFP complex appeared to be an appropriate sensor system for probing apoptosis [1,5].

In comparison to cell monolayers, multicellular tumor spheroids (MCTS) [6,7] appear more suitable to describe the situation in tissue and, in particular, in tumors in view of their physiology, cell-cell contacts and nutrient supply. Therefore, we now established 3-dimensional spheroids of HeLa cervical carcinoma cells expressing the Mem-ECFP-DEVD-EYFP sensor for caspase-3. Apoptosis was induced by the well-known toxin staurosporine as well as by phorbol-12-myristate-13-acetate (PMA) [8], an activator of protein kinase c, used in chemotherapy of cancer. In addition to microspectral analysis, fluorescence lifetime imaging (FLIM) was used to probe non-radiative energy transfer, and for this purpose a FLIM setup was combined with light sheet based fluorescence microscopy [9,10] in order to select individual planes of the MCTS. For a comparison and test of our method, the peptide linker DEVD was replaced by DEVG, which was less sensitive to caspase-3, thus maintaining a non-cleavable Mem-ECFP-DEVG-EYFP complex.

## 2. Results

### 2.1. Microspectral Analysis

**Figure 2:**
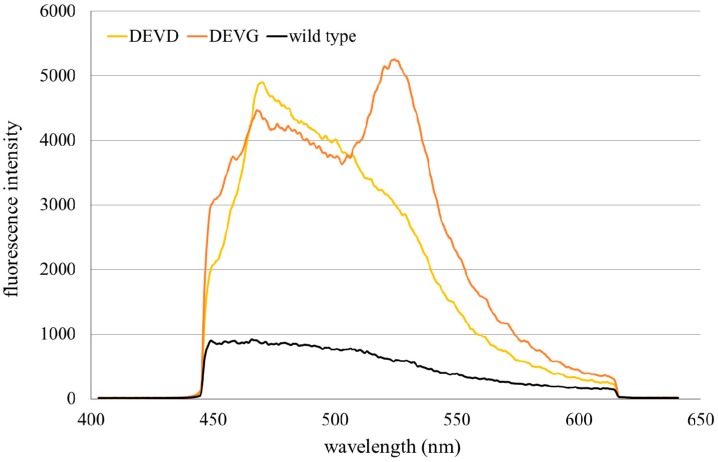
Fluorescence spectra of spheroids of HeLa cervical carcinoma cells transfected with the Mem-enhanced cyan fluorescent protein (ECFP)-DEVG-enhanced yellow fluorescent protein (EYFP) or the Mem-ECFP-DEVD-EYFP encoding vector in comparison with autofluorescence of non-transfected HeLa cells (“wild type”) after application of staurosporine (2 µM, 2.5 h); excitation wavelength: 405 nm.

Fluorescence spectra of HeLa cervical carcinoma cells transfected with the Mem-ECFP-DEVD-EYFP or with the Mem-ECFP-DEVG-EYFP encoding vector showed broad fluorescence bands around 470–480 nm as well as around 530 nm which previously have been assigned to ECFP and EYFP, respectively [1]. Since EYFP absorption is rather weak at the excitation wavelength of 391 nm, its strong fluorescence signal was mainly related to excitation via non-radiative energy transfer from ECFP. As depicted in Figure 2, multicellular spheroids containing the membrane associated complex ECFP-DEVG-EYFP still showed pronounced EYFP fluorescence after application of staurosporine (2 µM, 2.5 h), while cell spheroids transfected with the Mem-ECFP-DEVD-EYFP encoding vector showed almost no EYFP fluorescence upon incubation with this substance, thus proving that energy transfer was almost completely interrupted.

### 2.2. Light Sheet Based Fluorescence Microscopy and Lifetime Imaging (FLIM)

**Figure 3:**
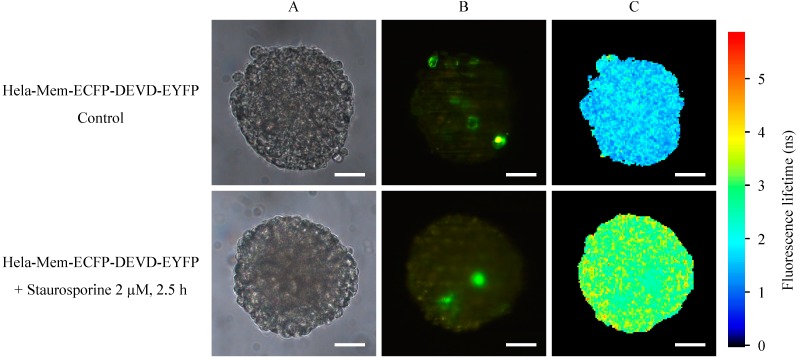
Transillumination (**A**), fluorescence intensity (λ ≥ 515 nm); (**B**) and fluorescence lifetime (450–490 nm); (**C**) of HeLa cervical carcinoma cells transfected with a Mem-ECFP-DEVD-EYFP encoding vector prior to and subsequent to application of staurosporine (2 µM, 2.5 h). Light sheet based fluorescence microscopy of individual layers of a multicellular spheroid with 6–10 µm thickness each. Excitation wavelength: 391 nm. Scale bars: 50 µm.

Spheroids of HeLa cervical carcinoma cells transfected with the Mem-ECFP-DEVD-EYFP encoding vector are depicted in Figure 3 prior to and subsequent to application of staurosporine (2 µM, 2.5 h). In addition to images upon transillumination (Figure 3A), fluorescence intensity (Figure 3B) and fluorescence lifetime images (Figure 3C) are depicted for light sheets of 6–10 µm thickness at an excitation wavelength of 391 nm. While in the fluorescence intensity images a wavelength range λ ≥ 515 nm is selected for detection of EYFP, a wavelength range of 450–490 nm is used for detection of fluorescence lifetime images of ECFP. Prior to application of staurosporine fluorescence of EYFP can be localized at the plasma membranes of individual cells, whereas subsequent to staurosporine application fluorescence of this protein is limited to the cytoplasm, possibly resulting from free EYFP molecules excited directly (without FRET). Fluorescence lifetime of the donor ECFP shows a pronounced prolongation (from an average value below 2 to about 3 nanoseconds) after application of staurosporine. This is related to cleavage of the DEVD linker and interruption of energy transfer to EYFP.

**Figure 4:**
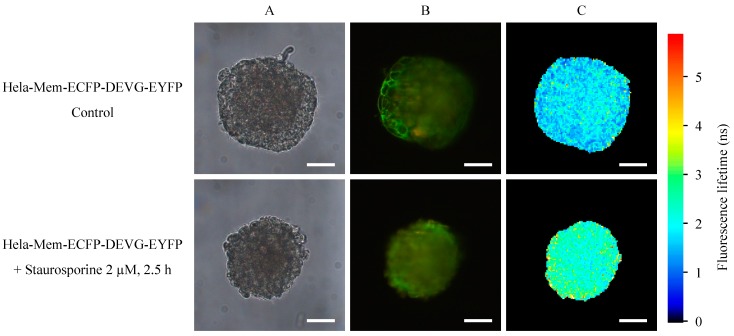
Transillumination (**A**), fluorescence intensity (λ ≥ 515 nm); (**B**) and fluorescence lifetime (450–490 nm); (**C**) of HeLa cervical carcinoma cells transfected with a Mem-ECFP-DEVG-EYFP encoding vector prior to and subsequent to application of staurosporine (2 µM, 2.5 h). Light sheet based fluorescence microscopy of individual layers of a multicellular spheroid with 6–10 µm thickness each. Excitation wavelength: 391 nm. Scale bars: 50 µm.

**Figure 5:**
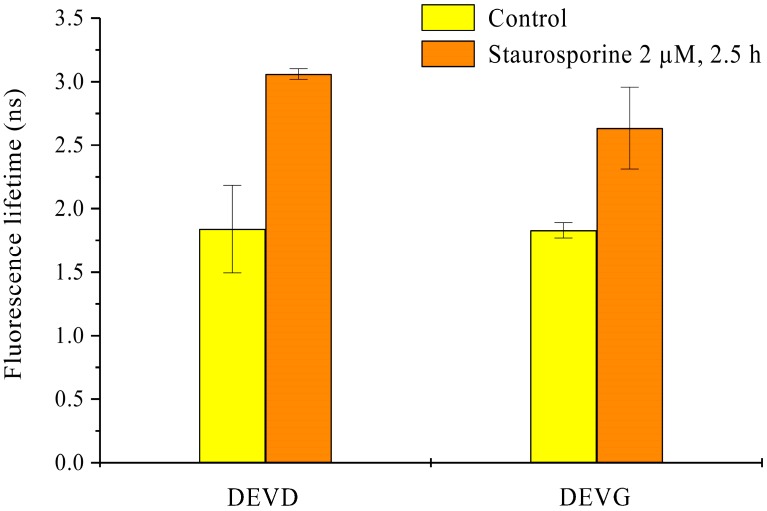
Fluorescence lifetime of the donor ECFP in transfected HeLa multicellular spheroids prior to and subsequent to application of staurosporine (2 µM, 2.5 h). Medians ± median absolute deviations (MADs) of six cell layers (from three spheroids) each selected by light sheet based fluorescence microscopy. Excitation wavelength: 391 nm; fluorescence detected at 450–490 nm).

Spheroids of HeLa cervical carcinoma cells transfected with the Mem-ECFP-DEVG-EYFP encoding vector are depicted in Figure 4 prior to and subsequent to application of staurosporine (2 µM, 2.5 h). Fluorescence intensity appears enhanced in comparison with Figure 3, probably due to a larger number of cells expressing the Mem-ECFP-DEVG-EYFP sensor. Similar to Figure 3, EYFP is located at the plasma membrane, but here some membrane location is maintained after incubation with staurosporine. Fluorescence lifetime appears only slightly prolonged by staurosporine application, and energy transfer from ECFP to EYFP is virtually maintained. A comparison of fluorescence lifetime of the donor ECFP in the Mem-ECFP-DEVD-EYFP as well as in the Mem-ECFP-DEVG-EYFP complex of HeLa cells prior to and subsequent to application of staurosporine is given in Figure 5. This Figure proves a pronounced prolongation of ECFP lifetime (medians from 1.8 to 3.0 ns) in the first case and a less pronounced prolongation of ECFP lifetime (medians from 1.8 to 2.5 ns) in the second case.

**Figure 6:**
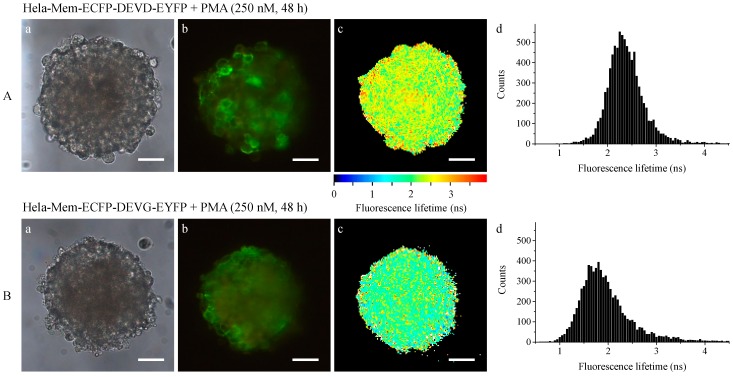
Transillumination (**a**), fluorescence intensity (λ ≥ 515 nm); (**b**) and fluorescence lifetime (450–490 nm); (**c**) including histograms; (**d**) of HeLa cervical carcinoma cells expressing Mem-ECFP-DEVD-EYFP (**A**) or Mem-ECFP-DEVD-EYFP (**B**) after application of phorbol-12-myristate-13-acetate (PMA) (250 nM, 48 h). Light sheet based fluorescence microscopy of individual layers of a multicellular spheroid with 6–10 µm thickness each. Excitation wavelength: 391 nm. Scale bars: 50 µm.

While apoptosis after application of staurosporine has been well documented for many years, cellular reactions upon application of pharmaceutical drugs may be more complex including necrosis and apoptosis. As an example we applied 250 nM phorbol-12-myristate-13-acetate (PMA) for 48 h to HeLa multicellular spheroids transfected with the Mem-ECFP-DEVD-EYFP or the Mem-ECFP-DEVG-EYFP vector. Similar to the Figure 3 and Figure 4 images of transillumination (a), fluorescence intensity of the acceptor EYFP (recorded at λ ≥ 515 nm); (b) and fluorescence lifetime of the donor ECFP (recorded at 450–490 nm); (c) are depicted in Figure 6. In addition, histograms are given to show the distribution of fluorescence lifetimes. Starting again at a fluorescence lifetime of about 1.8 ns before application of the drug, this lifetime remained almost the same after application of PMA to cells expressing the Mem-ECFP-DEVG-EYFP complex, but increased to an average value of about 2.4 ns after application to cells expressing the Mem-ECFP-DEVD-EYFP complex. The broad distribution of fluorescence lifetimes in both histograms indicates that part of the complexes may have been cleaved while other ones were probably maintained upon application of PMA. This is supported by the fluorescence of EYFP, part of which arises from the membranes of either cell line.

## 3. Discussion

A FRET based membrane associated sensor for apoptosis originally established in monolayers of HeLa cervical carcinoma cells [1] was now applied to multicellular tumor spheroids (MCTS). Upon optical excitation of enhanced cyan fluorescent protein (ECFP) non-radiative energy transfer (FRET) occurred to yellow fluorescent protein (EYFP) via the peptide linker DEVD. DEVD was cleaved upon apoptosis by caspase-3, and energy transfer was interrupted, resulting in a strong reduction of the fluorescent band of EYFP and a prolongation of the fluorescence decay time of ECFP, according to the equation:

1/τ − 1/τ_0_ = k_ET_ (1)

with k_ET_ corresponding to the energy transfer rate, τ to fluorescence lifetime in presence and τ_0_ to the fluorescence lifetime in absence of energy transfer. It should, however, be emphasized that fluorescence decay kinetics of ECFP were virtually bi-exponential with lifetimes of about 0.6 and 2.6 ns (in the presence of energy transfer), and that the fluorescence lifetimes depicted in the Figure 2, Figure 3, Figure 4 and Figure 5 represent so-called “effective lifetimes” τ_eff_ which would result from a mono-exponential fit of fluorescence decay curves. Nevertheless, it appeared that τ_eff_ was a fairly good measure to describe the impact of caspase-3 upon apoptosis in cell systems expressing the Mem-ECFP-DEVD-EYFP sensor. τ_eff_ increased significantly upon application of staurosporine, a well-known inducer of apoptosis, and increased moderately upon application of PMA which might initiate or enhance multiple reactions including apoptosis and necrosis. It remains to be further examined why a moderate increase of τ_eff_ occurred also in the Mem-ECFP-DEVG-EYFP complex, which was reported to be non-cleavable by caspase-3.

While total internal reflection fluorescence microscopy (TIRFM) was an appropriate method for probing apoptosis within the membranes of cell monolayers [1], light sheet based fluorescence microscopy appears to be the method of choice for characterizing 3D multicellular spheroids [10]. In contrast to laser scanning microscopy [11,12,13] and structured illumination microscopy [14,15] only those planes of the sample are exposed to light, which are examined simultaneously. Although in the present case upon illumination of up to 40 images with 1 J/cm^2^ the light exposure of individual planes exceeded the limit of non-phototoxic light doses [16], most other parts of the sample were not exposed to light, so that damage of the whole spheroid was not likely to occur. Obviously, as depicted in the Figure 2, Figure 3 and Figure 5, the fluorescence lifetime of ECFP and its increase upon apoptosis was homogenous within individual cell layers and varied only little within the whole spheroid. This proves that staurosporine and PMA had a similar impact on all parts of the spheroid and shows that the present sensor system may be appropriate for 3D applications.

So far, multicellular tumor spheroids (MCTS) proved to be appropriate 3-dimensional test systems for various substances, e.g., pharmaceutical agents or drugs. In the future, MCTS expressing the Mem-ECFP-DEVD-EYFP sensor may also be applied for validation of label free detection methods with some clinical or pre-clinical potential, e.g., autofluorescence, elastic light scattering with angular or spectral resolution as well as Raman spectroscopy.

## 4. Experimental Section

**Figure 7:**
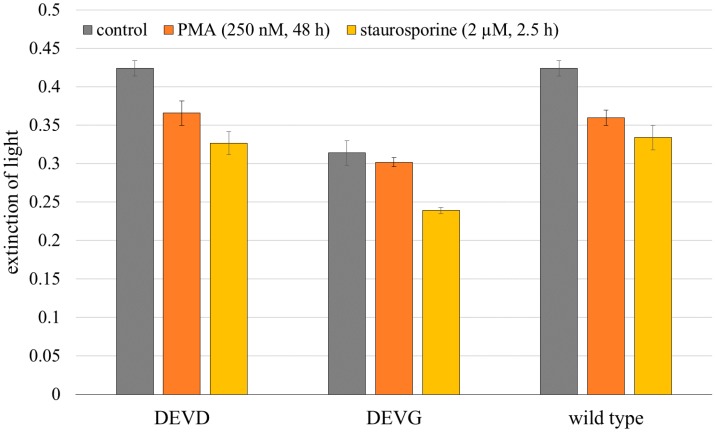
MTT (3-(4,5-dimethylthiazol-2-yl)-2,5-diphenyltetrazolium bromide) assay of HeLa cells without any treatment (control) and subsequent to application of staurosporine (2 µM, 2.5 h) or PMA (250 nM, 48 h). Medians ± median absolute deviations (MADs) of optical density readouts were determined after three independent measurements at 550 nm (background wavelength: 630 nm).

Materials—HeLa cervical carcinoma cells were grown at 37 °C, 5% CO_2_, in Minimum Essential Medium (MEM) supplemented with 10% FBS, non-essential amino acids, 100 U/mL penicillin, 100 ng/mL streptomycin and 2 mM glutamine. For cultivation of the stably transfected cell lines HeLa-Mem-ECFP-DEVD-EYFP and HeLa-Mem-ECFP-DEVG-EYFP G418 (200 µg/mL) was added to the culture medium. Multicellular spheroids (MCTS) were grown for 7–9 days up to a diameter of about 200 µm after seeding 150 transfected or 50 non-transfected cells per well in agarose gel–coated 96-well plates in appropriate culture medium at 5% CO_2_ and 37 °C. To induce apoptosis, cell spheroids were incubated within the microtiter plates with staurosporine (2 µM; 2.5 h) or PMA (250 nM; 48 h) dissolved in culture medium prior to transfer to the micro-capillary. For light sheet based fluorescence microscopy as well as for fluorescence spectroscopy cell spheroids were transferred into rectangular glass capillaries of 600 µm inner diameter using a pipette. A MTT (3-(4,5-dimethylthiazol-2-yl)-2,5-diphenyltetrazolium bromide)-assay was used to determine cytotoxicity following treatment with staurosporine or PMA. In healthy cells MTT is reduced to formazan dyes, giving a purple color [17]. 150 cells per well were plated in a 96-well plate in appropriate culture medium at 5% CO_2_ and 37 °C and grown for 5 days. After incubation with staurosporine or PMA cells were incubated with MTT (5 mg/mL) for 2 h at 5% CO_2_ and 37 °C. Afterwards, the cells were solubilized with DMSO (100 µL/well) and the optical density was measured in an ELISA Reader (MR 700 MICROPLATE READER, Dynatech Laboratories, Inc., Staffordshire, UK) at 550 nm (background wavelength: 630 nm). The results of this test are depicted in Figure 7 proving that the viability of the cells after treatment with staurosporine and PMA was clearly reduced.

Experimental setup—A laser diode (λ = 405 nm) was adapted to a fluorescence microscope (Axioplan 1, Carl Zeiss, Jena, Germany) by fiber optics for epi-illumination of whole cells. Fluorescence spectra of MCTS were recorded with a custom made polychromator and an image intensifying detection unit (IMD 4562, Hamamatsu Photonics, Ichino-Cho, Japan) at a resolution of 10 nm. A long pass filter for λ ≥ 420 nm was used.

**Figure 8:**
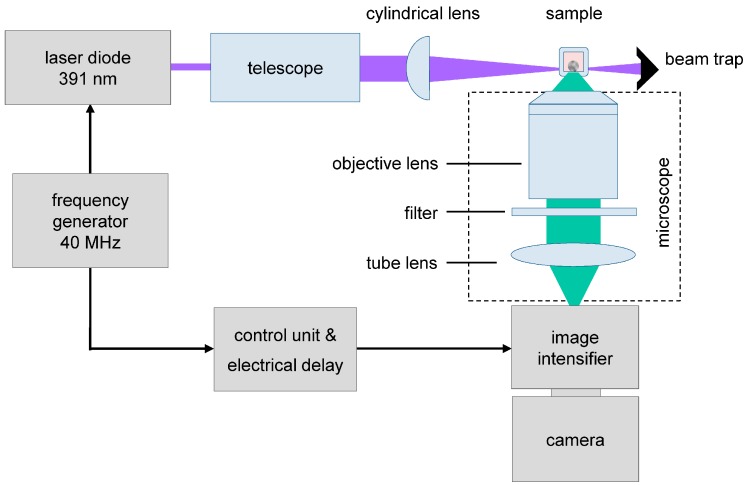
Fluorescence lifetime imaging (FLIM) using a light sheet based fluorescence microscope. Picosecond laser pulses and time-gated image detection are synchronized at a frequency of 40 MHz. All images are integrated over a period of 4 s.

A light sheet module for an inverse microscope (Axiovert 200, Carl Zeiss, Germany), as reported previously [10], was used to excite single planes of the cell spheroids with 6–10 µm thickness at an irradiance of about 200 mW/cm^2^. Samples were excited at 391 nm by a picosecond laser diode (LDH 400, PicoQuant, Berlin, Germany); fluorescence images of the acceptor (EYFP) were recorded at λ ≥ 515 nm by a CCD camera (AxioCam MRc, Carl Zeiss Microscopy GmbH, Jena, Germany), whereas fluorescence lifetime images of the donor (ECFP) were measured at 450–490 nm by an image intensifying camera system (Picostar HR, LaVision Biotec GmbH, Bielefeld, Germany). In this latter case a time gate of 200 ps was scanned over an axis of 8 ns, and the effective fluorescence lifetime was determined by a monoexponential fit of 40 images each (Figure 8). Transillumination images of whole spheroids were recorded for a comparison.
